# Xanthogranulomatous pyelonephritis: a focus on microbiological and antibiotic resistance profiles

**DOI:** 10.1186/s12894-021-00800-z

**Published:** 2021-04-07

**Authors:** A. Artiles-Medina, I. Laso-García, J. Lorca-Álvaro, M. Mata-Alcaraz, G. Duque-Ruiz, M. Hevia-Palacios, F. Arias-Funez, F. J. Burgos-Revilla

**Affiliations:** Department of Urology, Hospital Universitario Ramón Y Cajal. Instituto Ramón Y Cajal de Investigación Sanitaria (IRYCIS), Madrid, Spain

**Keywords:** Xanthogranulomatous pyelonephritis, Antibiotic resistance, Bacterial profile

## Abstract

**Background:**

Xanthogranulomatous pyelonephritis (XGP) is an inflammatory condition of the kidney and its treatment most often involves a combination of antibiotics and nephrectomy. This study aimed to define the clinical features and management of XGP, focusing on microbiological aspects and antibiotic therapy.

**Methods:**

We performed a retrospective study of 27 cases of XGP diagnosed between January 2001 and January 2020 to analyse their clinical and management characteristics. In addition, a literature review was conducted of XGP case series covering the period from 2000–2020. We searched PubMed for case series through April 2020 without language restrictions. Studies reporting case series of XGP (more than ten cases) were included if they were relevant to this study.

**Results:**

Twenty-seven patients were diagnosed with XGP, and 26 of them were histologically proven to have XGP. A total of 81.5% of the patients were female and the mean age was 59.6 years (SD 19.2). The most frequent symptoms were flank pain (70.4%) and fever (59.3%), while 77.8% of patients had renal stones. *Proteus mirabilis* was detected in the urine culture in 18.5% of patients, followed by detection of *Escherichia coli* in 14.8% of patients. The computed tomography (CT) findings included perirenal (29.6%) or pararenal (29.6%) involvement in the majority of patients. Twenty-six patients underwent nephrectomy. Piperacillin/tazobactam and ceftriaxone were the most commonly prescribed antibiotics for treatment. The reported piperacillin/tazobactam and ceftriaxone resistance rates were 14.3% and 16.6%, respectively. Twenty-six case series were included in the literature review, reporting 693 cases in total.

**Conclusion:**

We found well-established characteristics of XGP patients among series in terms of previous history, clinical, laboratory and imaging findings, and operative and postoperative outcomes. It is important to know the clinical presentation and potential severity of XGP, as well as the most frequently involved microorganisms and their antibiotic resistance profiles, to select the most appropriate antibiotic therapy.

**Supplementary Information:**

The online version contains supplementary material available at 10.1186/s12894-021-00800-z.

## Background

Xanthogranulomatous pyelonephritis (XGP) is an infrequent form of chronic granulomatous inflammation characterized by destruction of the renal parenchyma and replacement by solid sheets of lipid-laden macrophages. This rare entity constitutes less than 1% of chronic pyelonephritis [1]. The first case was reported by Schlagenhaufer in 1916. Because of diagnostic difficulties, relatively few cases have been published [2]. Since then, few series of cases (approximately 47 case series of at least ten reported cases) have been published, but there are no reviews compiling these series.

*Escherichia coli* and *Proteus mirabilis * are the isolated agents in approximately 90% of positive urinary cultures of XGP patients, although sterile urine cultures are not uncommon [3]. Generally, treatment of XGP includes antibiotic therapy and drainage of the abscess followed by nephrectomy for definitive treatment. Total nephrectomy (or in rare circumstances of focal XGP, partial nephrectomy) is the treatment of choice, and it is usually technically difficult due to extensive kidney adhesions to surrounding structures [4].

Antibiotics are used in all cases, but medical management only is rarely enough to solve the infection. Although surgery is regarded as the gold standard, antibiotic treatments have been proposed in children with unusual bilateral and focal XGP (with functioning kidney) [5]. In addition, symptomatic management through percutaneous drainage of abscesses can contribute to successful initial management of XGP.

Because XGP symptoms can be nonspecific, a diagnosis is often delayed, so imaging studies (ultrasound scan and computed tomography) are important in patients with recurrent or persistent pyelonephritis despite having been treated with adequate antibiotic therapy [6].

We report our case series (27 patients), focusing on fundamental, and yet insufficiently explored, microbiological and management aspects of XGP.

XGP can be a life-threatening condition; thus, data about culture isolates and antimicrobial resistances may guide antibiotic treatment in these cases. Our study contains antibiotics prescribed and antibiotic resistances identified in 27 cases of XGP, which are aspects not previously reported in the literature.

## Methods

A review of discharge records from the urology department was conducted to identify patients with a diagnosis of XGP between January 2001 and January 2020 at our institution.

This is a retrospective cross-sectional study in which we collected data about clinical manifestations (symptoms and physical examination findings), risk factors, radiologic and laboratory (including microbiological) findings, and details of the management and the postoperative period. We analysed urine culture isolates, detected antibiotic resistance and used antimicrobial therapy. Our laboratory used a threshold of 10^5^ CFU/ml as a positive urine culture during the study period. Data sources were electronic medical records and nephrectomy specimens from the records of the Department of Pathology.

An extensive literature review was conducted of XGP case series covering the period from 2000 to 2020. This period was established to avoid biases due to diagnostic accuracy and management strategies; therefore, we selected only modern case series, similar to our series (2001–2020). The search strategy used in PubMed was as follows: "Pyelonephritis, Xanthogranulomatous"[Mesh] OR "Xanthogranulomatous Pyelonephritis"[Title/abstract]. No language restriction was applied. Case series were included in the analysis if they reported more than ten cases and if they had information on the following variables (at least five): mean patient age, sex, risk factors (diabetes mellitus, immunosuppression, urolithiasis), clinical presentation, concomitant tumour, CT findings, main pathogens, prescribed antibiotics, nephrectomy approach and surgical complications.

## Results

Between 2001 and 2020, 27 patients were diagnosed with XGP and 26 of them were histologically proven to have XGP (one patient refused nephrectomy). Table 1 shows the patients’ demographic characteristics and clinical presentation.

**Table 1:** Demographic characteristics and symptoms

Variable	Value
Age in years, mean (SD)	59.6 (19.2)
Female vs male, n (%)	22 (81.5%) vs 5 (18.5%)
*Side*	
Right kidney, n (%)	14 (51.9%)
Left kidney, n (%)	13 (48.1)
DM, n (%)	3 (11.1%)
Immunosuppression, n (%)	2 (7.4%)
Concomitant renal tumour, n (%)	1 (3.7%)
Nephrolithiasis	21 (77.8%)
Staghorn, n (%)	19 (90%)
Size in mm, mean (SD)	23.5 (4.7)
*Localization, n (%)*	
Multiple	17 (81%)
Renal pelvis	3 (14.3%)
Upper calyx	1 (4.8%)
Ureteric stones, n (%)	3 (11.1%)
Hydronephrosis, n (%)	17 (63%)
Recurrent urinary tract infections (UTI), n (%)	9 (33.3%)
*Clinical presentation*	
Acute debut, n (%)	17 (63%)
Renal mass, n (%)	7 (25.9%)
Gross hematuria, n (%)	1 (3.7%)
Flank pain, n (%)	19 (70.4%)
Constitutional syndrome, n (%)	3 (11.1%)
Weight loss, n (%)	3 (11.1%)
Fever, n (%)	16 (59.3%)

DM: diabetes mellitus

Table 2 summarizes the laboratory, radiologic and microbiological findings. Urine culture was positive in 13 cases (48.1%). *P. mirabilis* was detected in the urine culture in 18.5% of patients, followed by detection of *E. coli* in 14.8% of patients. A mixed infection was detected in 2 patients (7.4%).

**Table 2:** Laboratory, radiologic and microbiological findings

Variable	Value (n and %)
**Laboratory**	
*Urine sediment*	
Pyuria, n (%)	7 (25.9%)
Microscopic hematuria, n (%)	3 (11.1%)
*Blood tests*	
Hemoglobin in g/dL, mean (SD)	10.1 (SD 1.5)
Serum creatinine in mg/dL, mean (SD)	1.5 (SD 1.2)
Estimated GFR in mL/min, mean (SD)	62.01 (SD 27.39)
WBC count per microliter, mean (SD)	12,508.1 (SD 4350.1)
**CT findings (M&E classification)**	
Malek 1	10 (37%)
Malek 2	8 (29.6%)
Malek 3	8 (29.6%)
Psoas abscess	3 (11.1%)
**Positive urine culture**	13 (48.1%)
* Proteus mirabilis*	5 (18.5%)
* Escherichia coli*	4 (14.8%)
* Pseudomonas aeruginosa*	2 (7.4%)
Mixed	
* E. coli* + *Streptococcus constellatus*	1 (3.7%)
* P. mirabilis* + *Serratia marcescens*	1 (3.7%)
**Prescribed antibiotics**	
Piperacillin/tazobactam (TZP)	13 (48.1%)
Ceftriaxone	5 (18.5%)
Amoxicillin/clavulanic acid	3 (11.1%)
Others	6 (21.9%)
**Overall antibiotic resistance profile (%)**	
Quinolones	14.30%
Carbapenems	0%
Amoxicillin/clavulanic acid	12.50%
Piperacillin/tazobactam (TZP)	14.30%
Ceftriaxone	16.60%
Fosfomycin	33.30%
Trimethoprim/sulfamethoxazole (TMP/SMX)	33.30%

GFR: glomerular filtration rate

The computed tomography (CT) findings included perirenal (29.6%) or pararenal (29.6%) involvement in the majority of patients and revealed a psoas abscess in three patients (11.1%).

Piperacillin/tazobactam (TZP) was the most commonly used antibiotic to treat XGP (48.1%) and ceftriaxone, a third-generation cephalosporin, was the second most prescribed antibiotic (18.5%). However, 14.3% of isolates exhibited some level of resistance to TZP and 16.6% were resistant to ceftriaxone.

Table 3 contains surgical data and postoperative complications of our case series. An open approach was used in 96.1% of the cases. Two patients had intestinal lesions and required a second operation (Clavien 3b). Two patients had septic shock and died in the early postoperative period (Clavien 5). Two patients needed blood transfusion and one patient had acute decompensated heart failure (Clavien 2). Another patient had a life-threatening pneumothorax that required drainage and ventilator support in the intensive care unit (Clavien 4b). Finally, surgical site infection was observed in 2 patients (Clavien 1). The overall complication rate was 38.5%.

**Table 3:** Surgical data and complications

Variable	Value (n and %)
Nephrectomy, n (%)	26 (96.3%)
*Approach*	
Open	25 (96.1%)
Laparoscopic	1 (3.8%)
*Type of procedure*	
Urgent	9 (34.6%)
Elective or non-urgent	17 (65.4%)
Time-to-surgery in days (from clinical suspicious), mean (SD)	38.2 (54.5)
*Postoperative complications (Clavien Dindo classification)*	10 (38.5%)
Grado 1	2 (20%)
Grado 2	3 (30%)
Grado 3a	0
Grado 3b	2 (20%)
Grado 4	1 (10%)
Grado 5	2 (20%)
Overall mortality	2 (7.4%)

The supplementary table (see Additional file 1) summarizes case series between 2000 and 2020 [23-45]. Twenty-six case series met the inclusion criteria and were included, reporting 693 cases. Out of 26 case series, the most frequent isolate was *E. coli* in 8 of the series. After data extraction, we only identified one case series that reported the antibiotic prescribed.

## Discussion

XGP is a rare, severe, chronic inflammatory condition of the kidney that leads to a nonfunctioning enlarged kidney and is normally associated with obstructive uropathy secondary to nephrolithiasis [7]. This entity is increasingly well recognized and its confirmatory diagnosis is based on histopathological examination [1]. Only a few case series have been published.

**Fig. 1:**
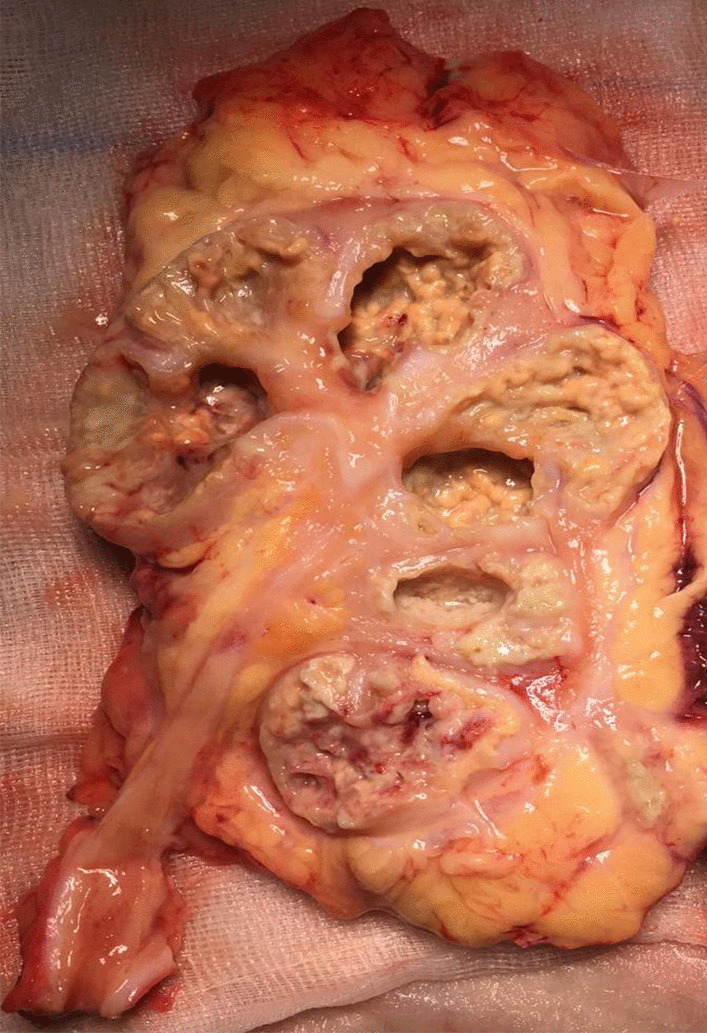
Gross examination shows enlarged kidney and yellow nodules in the renal parenchyma

XGP, a so-called “inflammatory tumour” of the kidney, involves the calyces, renal parenchyma and renal sinus areas, and it may spread into the perinephric tissue with the formation of abscesses and even fistulas [8] (Fig. 1). Its pathogenesis remains unclear, although it seems that bacterial infection and urinary obstruction are important factors in its pathophysiology. Histologically, xanthogranulomatous pyelonephritis consists of a granulomatous inflammatory infiltrate composed of neutrophils, lymphocytes, plasma cells, xanthomatous histiocytes, and multinucleated giant cells [9].

A correct preoperative diagnosis of XGP is very difficult because this entity can mimic other disorders, such as renal neoplasms (including clear cell renal cell carcinoma, papillary renal cell carcinoma, sarcomatoid renal cell carcinoma, leiomyosarcoma, Wilms tumour), chronic pyelonephritis, nephrolithiasis, tuberculosis, malakoplakia and megalocytic interstitial nephritis. It is well known that preoperative diagnosis of XGP is difficult, but high-resolution imaging techniques have enhanced diagnostic accuracy [10]. Studies have reported that a preoperative diagnosis can be achieved through a combination of clinical and radiologic data in only 40% of cases [11]. Generally, this chronic inflammatory process is unilateral, most frequently affects middle-aged women, and occasionally manifests in childhood.

Symptoms include flank pain, weight loss, and fever. Regarding clinical presentation, Al-Ghazo et al. reported a series of 18 cases and noted that the clinical characteristics of XGP included calculi or obstruction in the urinary tract, damage to the kidney, complications of urinary tract infection, anaemia and increased erythrocyte sedimentation rate. Extended infections, such as psoas abscess, nephrocutaneous fistula, renocolonic fistula and paranephric abscess, can be found in the third group of cases [12]. Similarly, in our retrospective study, the computed tomography (CT) findings included perirenal (29.6%) or pararenal (29.6%) involvement in the majority of patients and a psoas abscess in three cases (11.1%).

Kundu et al. conducted a 13-year retrospective study (2005–2017) to describe the clinicopathologic spectrum of XGP. This author described forty cases of XGP and reported diffuse renal parenchymal involvement in the majority of patients (77.5%) and a high rate (90%) of nephrolithiasis. Furthermore, in their series, these authors identified 5% of coexisting renal cell carcinomas in the same kidney [13]. In our case series, we found a nephrolithiasis rate of 77.8% and only one patient presented with concomitant renal cell carcinoma (3.7%).

Based on published cases, the characteristics of our series are similar to those that have been previously reported.

**Fig. 2:**
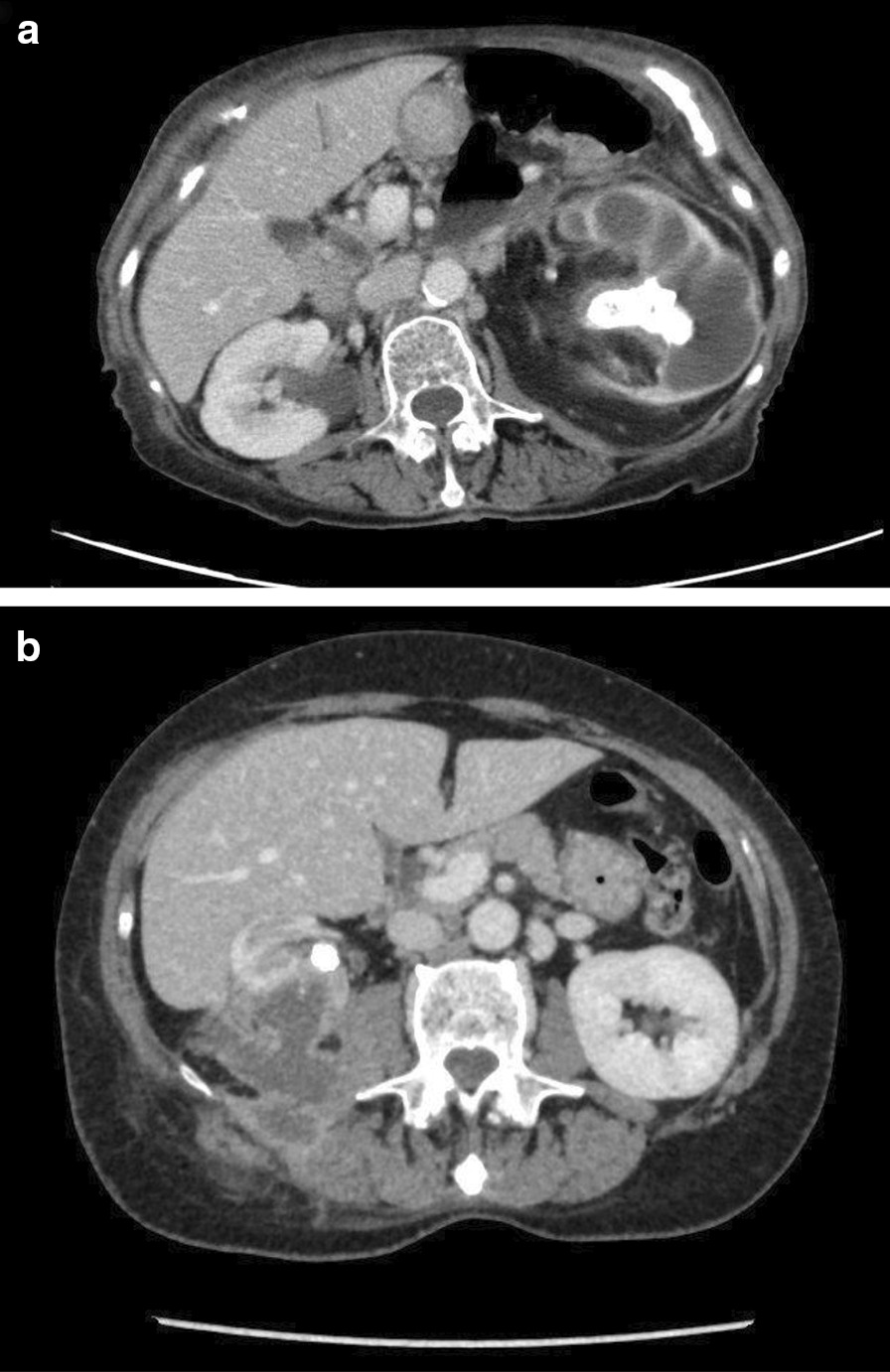
Axial CT scan images demonstrate obstructing left pelvic staghorn stone with associated grade IV hydronephrosis (Malek I) (**a**) and retroperitoneal collections surrounding the right kidney with a renal pelvic calculus (Malek III) (**b**)

Computed tomography is the mainstay of diagnostic imaging for xanthogranulomatous pyelonephritis. Eastham et al. retrospectively reviewed all medical records, including radiographic materials, of 27 patients with a pathologic diagnosis of XGP and reported an accuracy of 87% for CT scans for the preoperative diagnosis of XGP. CT accurately defined the extent of the perinephric inflammatory reaction [14]. According to Malek and Elder’s radiological classification, XGP can be subdivided into 3 stages on the basis of the extent of involvement of the adjacent tissue [15]: stage I, nephric disease confined to the kidney; stage II, infiltration into the Gerota fascia; and stage III, disease extending into adjacent perinephric space and other retroperitoneal structures (Fig. 2) [16].

There are scarce data on antibiotic treatment used in XGP. It has been reported that one-third of patients have sterile urine and one-fourth have mixed growth in urine culture. The most frequent microorganisms isolated in urine culture are *P. mirabilis*, followed by *E. coli*. In the study of Dwivedi et al., these microorganisms were isolated in 23% and 11.5% of cases, respectively [7].

Petca et al. conducted a retrospective observational study from January 2013 to December 2017 that identified 29 patients diagnosed with XGP, of which 18 patients (62.06%) showed a positive urine culture. In 16 out of the 18 cases with positive urine culture, a gram-negative microorganism from the *Enterobacteriaceae* family was isolated, while gram-positive microorganisms were detected in only two cases (6.89%). Moreover, they reported that the microorganism most commonly identified was *E. coli*, followed by *Proteus, Klebsiella, Enterococcus* and *Staphylococcus *[17]. Consistent with this author, we reported a positive urine culture rate of 48.1%. However, in our study *P. mirabilis* was the most common microorganism detected and was found in the urine culture in 18.5% of patients, followed by detection of *E. coli* in 14.8% of patients. A mixed infection was detected in 2 patients (Table 2).

Conservative management of XGP (with antibiotics or partial nephrectomy) has been described for focal forms of the disease. Nevertheless, diffuse forms usually require total nephrectomy [18]. As recommended by Schlossberg et al., extended-spectrum penicillin (e.g., piperacillin–tazobactam), cephalosporin (ceftriaxone or cefotaxime), fluoroquinolone (ciprofloxacin) or ampicillin with gentamicin are all appropriate choices for treatment. Intravenous treatment for 24–48 h after resolution of symptoms and fever is necessary, followed by at least 2 weeks of oral antibiotics based on susceptibility testing [19]. In our case series, piperacillin–tazobactam (48.1%) and ceftriaxone (18.5%) were the most frequent antibiotic therapies.

Considering increasing antibiotic resistance rates over the last decades, antibiotic therapy recommendations and prescribing patterns for acute pyelonephritis have changed. *Enterobacteriaceae* are common uropathogens causing acute pyelonephritis (APN), and *E. coli* is the most frequent among them. These uropathogens have become resistant to several important antibiotics, such as trimethoprim/sulfamethoxazole, fluoroquinolones (FQs) and 3^rd^ generation cephalosporins (3^rd^ CEPs). According to a study conducted in Korea by Kim et al., 3^rd^ CEPs were the most commonly prescribed antibiotics (41.4%) for inpatients, followed by FQs. However, the use of 3^rd^ CEPs, beta-lactam/beta-lactamase inhibitors, and carbapenems increased substantially for the treatment of hospitalized APN patients. In particular, carbapenem use increased 3.1-fold over 5 years [20]. Moreover, the Study for Monitoring Antimicrobial Resistance Trends (SMART), which has collected 1,116 gram-negative urinary pathogens from hospitalized patients in 33 countries during 2009–2010, showed that the rates of fluoroquinolone resistance (FQR) in gram-negative bacilli vary widely country to country; for example, they were 23.5% in North America, 29.4% in Europe, and 33.2% in Asia. As a consequence, this study suggests that FQs may no longer be effective as first-line therapy for gram-negative UTIs in hospitalized patients [21].

The use of the laparoscopic approach for surgical treatment of XGP is controversial, but according to published case series, it can be successfully performed. Nevertheless, a high conversion rate (approximately 27%) has been reported [4]. According to Khaira et al., laparoscopic nephrectomy in patients with XGP is often challenging and requires considerable experience in laparoscopy [22]. These authors reported grade 2 (Clavien-Dindo) complications in 40% and 29.4% of patients in the open and laparoscopic nephrectomy groups, respectively. In our study, the postoperative complication rate was 38.5% (grades 1–2 in 50% of complications).

Regarding the strengths and limitations of this study, we were able to include 27 patients diagnosed with a very rare disease. Our findings were consistent with prior studies. We were also able to analyse microbiological aspects, such as antibiotic resistance and antibiotic therapies, that are very relevant in this infectious process but they have been insufficiently addressed in the literature. Nevertheless, in our data set, there were some variables with missing values, as well as incomplete data for some patients.

## Conclusions

Xanthogranulomatous pyelonephritis is a rare entity, and its management usually involves antibiotics and total nephrectomy and occasionally drainage of abscesses. It is important to know its clinical presentation and potential severity, as well as the most frequently involved microorganisms and their antibiotic resistance profile, to select the most appropriate antibiotic therapy.

The literature review shows well-established characteristics of XGP patients among series in terms of previous history, clinical, laboratory and imaging findings, and operative and postoperative outcomes.

## Supplementary Information

**Additional file 1**. Supplementary table: Case series of XGP (more than 10 cases).

## Data Availability

The datasets will be available from the corresponding author upon reasonable request.

## Abbreviations

CT: Computed tomography
DM: Diabetes mellitus
ID: Immunodeficiency
LN: Laparoscopic nephrectomy
ON: Open nephrectomy
XGP: xanthogranulomatous Pyelonephritis
