# Supplementary material for: Xanthogranulomatous pyelonephritis: a focus on microbiological and antibiotic resistance profiles
Source: BMC Urol. 2021 Apr 7;21:56. doi: 10.1186/s12894-021-00800-z (PMC8026091; doi:10.1186/s12894-021-00800-z)
Supplement: Supplementary file 1 — Additional file 1. Supplementary table: Case series of XGP (more than 10 cases). [file 12894_2021_800_MOESM1_ESM.docx]

**Supplementary table: Case series of XGP (more than 10 cases)**

| **Author** | **n** | **Age** | **Gender (F vs M)** | **DM** | **ID** | **Urolithiasis** | **Clinical presentation** | **Concomitant tumour** | **CT findings (Malek stage ≥ 2)** | **Main pathogens** | **Prescribed antibiotics** | **Nephrectomy approach** | **Postoperative complications (Clavien-Dindo)** |
| --- | --- | --- | --- | --- | --- | --- | --- | --- | --- | --- | --- | --- | --- |
| Campanario-Pérez et al. 2018(23) | 17 | 60 (13.3%) | 10 (58.2%)  vs 7 (41.8%) | 2 (28.6%) | 2 (28.6%) | 13 (76.5%) | Fever: 6 (42.9%)  Pain: 4 (28.6%) | 2 (28.6%) | 10 (66.7%) | E. coli: 4 (66.7%)  Klebsiella: 1 (16.7%) | Not available | LN: 15 (88.2%)  ON: 2 (12.5%) | 6 (40%)  1: 1  2: 4  5: 1 |
| [Ichaoui](https://pubmed.ncbi.nlm.nih.gov/?sort=pubdate&term=Ichaoui+H&cauthor_id=30430527) et al. 2018(24) | 42 | 50 (34-79) | 61% vs 39% | 6 (14%) | 1 (2%) | 38 (90%) | Fever: 27 (64%)  Pain: 40 (95%) | Not available | 6 (14%) | E. coli: 15 (36%)  P. mirabilis: 7 (17%) | Not available | ON: 100% | Not available |
| [Stoica](https://pubmed.ncbi.nlm.nih.gov/?sort=pubdate&term=Stoica+I&cauthor_id=29233628) et al. 2018^Ψ^(25) | 66 | 4.84 (1.1-14.81) | 1.35:1 | Not available | Not available | 53 (80%) | Fever: 35 (53%)  Pain: 40 (61%) | Not available | 52 (79%) | P. mirabilis: 28 (42.4%)  E. coli: 19 (28.7%) | Not available | ON: 63 | 15 (23 %) |
| [Çaliskan](https://pubmed.ncbi.nlm.nih.gov/?sort=pubdate&term=%C3%87aliskan+S&cauthor_id=27743436) et al. 2016(26) | 13 | 56 ± 16.09 | 6 (46.15)  vs 7 (53.84) | Not available | Not available | 7 (53.84% | Fever: 4 (30.77)  Pain: 7 (53.84) | Not available | Not available | E. coli: 1 (10%)  Sterile: 90% | Not available | ON: 100% | Not reported |
| [Addison](https://pubmed.ncbi.nlm.nih.gov/?sort=pubdate&term=Addison+B&cauthor_id=24661744) et al. 2015(27) | 35 | 46.6 (12–81) | 32 (91) vs 3 (9) | 11 (31.4) | Not available | 74.3% | Not available | Not available | Not available | E. coli: 16 (45.7%)  P. mirabilis: 7 (20.0%)  B. fragilis: 4 (11.4%) | Not available | ON: 100% | 24 (60%)  1: 3 (8.8)  2: 8 (23.5)  3a: 0 (0)  3b: 7 (20.6)  4a: 4 (11.7)  4b: 0 (0)  5: 2 (5.9) |
| [Datta](https://pubmed.ncbi.nlm.nih.gov/?sort=pubdate&term=Datta+B&cauthor_id=25935947) et al. 2014(28) | 18 | Not available | 13 vs 5 | 2 | Not available | 100% | Not available | Not available | Not available | Sterile: 100% | Not available | ON: 100% | Not relevant |
| Kim et al. 2013(29) | 21 | 52.1 (36–89) | 15 (71.4) vs 6 (28.6) | 11 (52.4) | Not available | 9 | Fever: 14 (66.7)  Pain: 19 (90.5) | Not available | Not available | E. coli: 5 (23.8)  P. mirabilis: 3 (14.2)  Klebsiella: 1 (4.8) | Not available | ON: 100% (1 PN) | Not available |
| Nam et al. 2012^Ψ^(30) | 14 | 79.4 ± 66.5 months | 5 vs 9 | Not available | Not available | Not available | Fever: 85.7%:  Pain: 57.1% | Not available | Not available | P. mirabilis: 4  E. coli: 1 | Not available | ON: 13 | Not available |
| [Lima](https://pubmed.ncbi.nlm.nih.gov/?sort=pubdate&term=Lima+M&cauthor_id=22948458)  et al. 2012(31) | 66 | LN: 41.5 ± 8.2  ON: 42.5 ± 9.6 | 39 vs 27 | Not available | Not available | 100% | Not available | Not available | Not available | Not available | Not available | LN: 58 (87.9%)  ON: 8 (12.1%) | ON: 1 major (colonic lesion) and 2 minor (wound infection) |
| Kuo et al. 2011(32) | 30 | 55.17 | 25 (83.3%) vs 5 | Not available | Not available | 56.7% | Not available | Not available | Stage 3: 33.3% | E. coli: 36.7% | Not available | Not available | Not available |
| **Author** | **n** | **Age** | **Gender (F vs M)** | **DM** | **ID** | **Urolithiasis** | **Clinical presentation** | **Concomitant tumour** | **CT findings (Malek stage ≥ 2)** | **Main pathogens** | **Prescribed antibiotics** | **Nephrectomy approach** | **Postoperative complications (Clavien-Dindo)** |
| [Guzzo](https://pubmed.ncbi.nlm.nih.gov/?sort=pubdate&term=Guzzo+TJ&cauthor_id=19389010) et al. 2009(33) | 27 | 43.5 (17–85) | 18 vs 8 | Not available | Not available | 17 (65%) | Not available | Not available | Not available | P. mirabilis: 6 (25)  E. coli: 4 (17)  *Staphylococcus*: 2 (8) | Not available | LN: 14 (54%)  ON: 12 (46%) | LN: 3  ON: 4 |
| [Korkes](https://pubmed.ncbi.nlm.nih.gov/?sort=pubdate&term=Korkes+F&cauthor_id=18308077) et al. 2008(34) | 41 | 47± 17 | 5 (85.4%) VS 6 (14.6%) | Not available | Not available | 100% | Not available | Not available | 22.0% | E. coli: 35.3%  P. mirabilis: 17.6%  Sterile: 35% | Not available | LN: 1  ON: 40 -standard flank incision 81.3% and 15.6% laparotomy | 21.9% |
| [Loffroy](https://pubmed.ncbi.nlm.nih.gov/?sort=pubdate&term=Loffroy+R&cauthor_id=17662737) et al. 2007(35) | 13 | 55.2 (30-87) | 10 vs 3 | 1 | Not available | 8 (72.7%) | Fever: 9 (69.2)  Anorexia with weight loss: 9 (69.2)  Pain: 7 (53.8). | Not available | 6 (54.5%) | E. coli: 3 (23.1%)  P. mirabilis: 2 (15.4%) | Not available | 100% ON: 12 total and 1 partial | Not available |
| [Vanderbrink](https://pubmed.ncbi.nlm.nih.gov/?sort=pubdate&term=Vanderbrink+BA&cauthor_id=17263611) et al. 2007(36) | 12 | LN: 54 ± 12  ON: 45.7 ± 33 | Not available | Not available | Not available | 6 (50%) | Not available | Not available | Not available | Not available | Not available | LN: 5 (1 conversion)  ON: 6 | LN: 3 (50%)  ON: 2 (33%) |
| [Kapoor](https://pubmed.ncbi.nlm.nih.gov/?sort=pubdate&term=Kapoor+R&cauthor_id=17113885) et al. 2006(37) | 25 | LN: 37.1 (16-61)  ON: 48.1 (10 -75) | LN: 4 vs 6  ON: 4 vs 11 | LN: 0  ON: 6 (40%) | Not available | LN: 5  ON: 3 | Pain: 60% ON and 70% LN  Fever: 53% ON and 60% LN | Not available | Not available | Not available | Not available | LN: 10 (2 conversion)  ON: 15 | LN: 1 (10%) major and 2 (20%) minor  ON: 4 (26.6%) minor |
| [Dwivedi](https://pubmed.ncbi.nlm.nih.gov/?sort=pubdate&term=Dwivedi+US&cauthor_id=17054551) et al. 2006(7) | 26 | 42 (6–65) | 10 vs 16 | Not available | Not available | Not available | Fever: 22 (84.61%)  Pain: 21 (80.76%) | Not available | Not available | E. coli: 3 (11.53)  P. mirabilis: 6 (23.07)  *Staphylococcus*: 1 (3.84)  Mixed: 6 (23.07) | Not available | 100% | 2 major |
| [Al-Ghazo](https://pubmed.ncbi.nlm.nih.gov/?sort=pubdate&term=Al-Ghazo+MA&cauthor_id=17098659) et al. 2006(12) | 18 | 50 (3–65) | 13 vs 5 | 4 (22.2%) | Not available | Renal: 14 (77.8%)  Ureteric: 1 (5.6%) | Pain: 17 (94.4%)  Fever: 5 (35.7%)  Weight loss: 15 (88.3%) | Not available | Not available | Positive culture: 16 (88.9%). P. mirabilis and E. coli: most common | Not available | 100% | 2 minor |
| [Rosoff](https://pubmed.ncbi.nlm.nih.gov/?sort=pubdate&term=Rosoff+JS&cauthor_id=17070338) et al. 2006(38) | 11 | 51 (28 -78) | 6 vs 5 | 4 | Not available | 7 | Not available | Not available | Not available | Not available | 100% ampicillin and gentamicin | 100% LN (4 conversion) | 4: 2 ileus, 2 persistent fever |
| **Author** | **n** | **Age** | **Gender (F vs M)** | **DM** | **ID** | **Urolithiasis** | **Clinical presentation** | **Concomitant tumour** | **CT findings (Malek stage ≥ 2)** | **Main pathogens** | **Prescribed antibiotics** | **Nephrectomy approach** | **Complications (Clavien-Dindo)** |
| [Nawaz](https://pubmed.ncbi.nlm.nih.gov/?sort=pubdate&term=Nawaz+H&cauthor_id=16302473) et al. 2005(39) | 63 | 11 - 70 | 37 vs 26 | 16 (25%) | Not available | Renal: 56 (88.8%)  Ureteric: 17 (26.9%) | Fever + pain: 93.6% | Not available | Not available | P. mirabilis: 21 (33%)  E. coli: 16 (25%)  Klebsiella: 10 (15.8%)  S. aureuss: 7 (8%) | Not available | 100% ON | Not available |
| [Khaira](https://pubmed.ncbi.nlm.nih.gov/?sort=pubdate&term=Khaira+HS&cauthor_id=16190834) et al. 2005(22) | 12 | ON: 54.4 (29-83)  LN: 42.5 (36-57) | Not available | Not available | Not available | ON: 5 (63)  LN: 3 (100) | ON: Pain 2 (25)  LN: Pain 1 (33) | Not available | Not available | Not available | Not available | ON: 8  LN: 3 (1 conversion) | ON: 5 (3 minor and 2 major)  LN: 1 |
| [Saavedra Jo](https://pubmed.ncbi.nlm.nih.gov/?sort=pubdate&term=Saavedra+Jo+S&cauthor_id=15382434) et al. 2004(40) | 11 | 45 (23-68) | 9 (82%) vs 2 (18%) | Not available | Not available | 70% | Renal mass: 91%  Pain: 82%  Fever: 64% | Not available | Not available | P. mirabilis: 40%  E. coli: 40%  Klebsiella: 10% | Not available | 100% ON: simple 6 and radical 5 | 4 (36%) |
| Zorzos et al. 2003(41) | 39 | 57 (24–85) | 25 females and 14 males | 4 | 1 | 7 | Commonest: fever, pain and malaise | Not available | Involvement of the posterior pararenal space (4), psoas muscle (5), diaphragm (2) and spleen (1) | Sterile: 55%  E. coli and P. mirabilis: most common | Not available | 100% ON | 8 wound infections, 1 duodenal fistula, and 1 retroperitoneal abscess |
| [Bingöl-Koloğlu](https://pubmed.ncbi.nlm.nih.gov/?sort=pubdate&term=Bing%C3%B6l-Kolo%C4%9Flu+M&cauthor_id=11967759) et al. 2002^Ψ^(42) | 17 | 6.6 ± 0.8 | 2 vs 15 | Not available | Not available | 9 (53 %) | Not available | Not available | Perirenal: 5  Psoas: 2 | P. mirabilis + E. coli + Klebsiella: 10 (59 %) | Not available | ON: total 14, partial 2 | Colonic perforation: 3  Wound infection: 3 |
| [Samuel](https://pubmed.ncbi.nlm.nih.gov/?sort=pubdate&term=Samuel+M&cauthor_id=11283885) et al. 2001^Ψ^(43) | 19 | 3.4 ± 1.7 | 8 vs 11 | Not available | Not available | Renal: 13 | Renal mass: 6  Fever: 6  Hematuria: 6 | Not available | Not available | P. mirabilis: 8 (62%)  E. coli: 2 (15%)  Mixed: 2 (15%) | Not available | ON: 12, partial 1 | Not available |
| [Tiu](https://pubmed.ncbi.nlm.nih.gov/?sort=pubdate&term=Tiu+CM&cauthor_id=11486322) et al. 2001(44) | 27 | 57 (21-86) | 15 vs 12 | Not available | Not available | Renal: 14 (52%)  Ureteral: 3 (11%) | Pain: 18 (67%)  Fever: 14 (52%) | Not available | Not available | E. coli and P. mirabilis: 16 (80%) | Not available | Not available | Not available |
| Kim et al. 2001(45) | 20 | 45 (3 – 61) | 16 vs 4 | Not available | Not available | 16 (76%) | Recurrent fever: 6  Dysuria: 7  Renal mass: 4 | Not available | Extrarenal extension: 13 (62%) | Not available | Not available | Not available | Not available |

^Ψ^ Pediatric patients
